# Evaluation of Metabolic Characteristics Induced by Deoxynivalenol in 3D4/21 Cells

**DOI:** 10.3390/ani15152324

**Published:** 2025-08-07

**Authors:** Yu Han, Bo Yu, Wenao Weng, Liangyu Shi, Jing Zhang

**Affiliations:** Laboratory of Genetic Breeding, Reproduction and Precision Livestock Farming & Hubei Provincial Center of Technology Innovation for Domestic Animal Breeding, School of Animal Science and Nutritional Engineering, Wuhan Polytechnic University, Wuhan 430023, China; hy13018038681@126.com (Y.H.); wonderfish@whpu.edu.cn (B.Y.); wenao15838719410@126.com (W.W.)

**Keywords:** deoxynivalenol, pig, alveolar macrophages, metabolomics

## Abstract

Deoxynivalenol (DON) is a common mycotoxin that causes immunosuppression in pigs. Its effects on cellular metabolism remain unclear. In this study, we investigate DON-induced metabolic alterations in porcine alveolar macrophage cell line 3D4/21 using non-targeted metabolomics. MTT assays showed DON reduced cell viability in a concentration- and time-dependent manner. Principal component analysis (PCA) and orthogonal partial least squares discriminant analysis (OPLS-DA) revealed distinct metabolic profiles between control and DON-treated groups. Metabolomic analysis identified 127 differential metabolites (VIP > 1, *p* < 0.05), primarily in purine metabolism, glutathione metabolism, and arginine–proline metabolism. Integration with transcriptomic data confirmed that these pathways play key roles in DON-induced immunotoxicity. Specifically, changes in purine metabolism suggested disrupted nucleotide synthesis and energy balance, while glutathione depletion indicated weakened antioxidant defense. These findings provided a systems biology perspective on DON’s metabolic reprogramming of immune cells and identified potential therapeutic targets to reduce mycotoxin-related immunosuppression in swine.

## Simple Summary

Deoxynivalenol (DON) is a common mycotoxin that weakens immune function in pigs. However, its effects on cellular metabolism remain unclear. This study employs porcine alveolar macrophages (3D4/21 cells) to investigate DON-induced metabolic alterations using non-targeted metabolomics. MTT assays showed DON reduced cell viability in a concentration- and time-dependent manner. Metabolomic analysis identified 127 differential metabolites, revealing distinct metabolic profiles between control and DON-treated cells. These changes mainly affected purine metabolism, glutathione metabolism, and arginine–proline metabolism. Integration with transcriptomics data confirmed these pathways are important for DON-induced immunotoxicity. The study provides new insights into DON-induced metabolic reprogramming in immune cells and identifies candidate targets for alleviating mycotoxin-driven immunosuppression in swine.

## 1. Introduction

Mycotoxin contamination in feed is a major threat to global livestock health and causes severe economic losses in agriculture [1]. Among these toxins, deoxynivalenol (DON), a type B trichothecene produced by *Fusarium* species, is one of the most widespread contaminants in cereal crops worldwide [2]. Due to its high stability during storage and food processing, DON exposure through contaminated feed is almost unavoidable in the diet of domestic animals [3]. Even at subclinical levels, DON exposure reduces feed intake, slows growth, and decreases feed conversion efficiency, further harming productivity [4].

Pigs are especially susceptible to DON toxicity owing to their substantial dietary consumption of DON-contaminated cereals (wheat, corn, and barley) [5]. Compared with other domestic animals, pigs have a higher absorption rate of DON (exceeding 70%), mainly due to the short transit time of feed through the gastrointestinal tract and the efficient absorption of DON in the upper small intestine [4,6]. Moreover, only a small amount of DON can be converted to the less toxic metabolite DOM-1, resulting in high bioavailability of the toxin in the pig’s bloodstream [7]. Consequently, pigs are particularly susceptible to DON toxicity, even at low exposure levels. Previous research shows that even low DON levels (3.02 mg/kg) can reduce feed intake and growth in pigs [5], while in vitro studies report that DON as low as 0.8 μM impairs porcine immune cell function [8].

DON has strong effects on the porcine immune system in multiple ways. It directly damages immune organs such as the thymus and spleen, leading to atrophy of the thymic cortex, reduction in lymphocyte populations, and impaired development of germinal centers in the spleen [9,10,11]. In pig models, DON exposure leads to thymic atrophy and increased apoptosis of thymocytes, with significant reductions in cortical lymphocyte density [10,12]. In addition, DON has been reported to impair both T-cell and B-cell function in porcine peripheral blood mononuclear cells (PBMCs) [8]. However, the global metabolic alterations underlying DON-induced immunotoxicity remain poorly characterized.

Metabolomics, a powerful systems biology tool, provides a comprehensive view of how xenobiotic exposure affects cellular metabolism [13]. Non-targeted metabolomic approaches are particularly advantageous for elucidating unexpected pathways altered by toxic insults [14,15]. By mapping these metabolic alterations, researchers can pinpoint specific biomarkers for early detection of xenobiotic exposure, evaluate the severity of its toxic effects, and understand interindividual variability in susceptibility [13,16]. Nevertheless, limited data exist on DON-driven metabolic dysregulation in porcine immune cells, especially in the context of alveolar macrophage functionality.

Since alveolar macrophages play a key role in the first line of defense against pulmonary pathogens, the porcine alveolar macrophage cell line 3D4/21 has emerged as a valuable in vitro model to investigate DON-induced cellular and molecular alterations in pigs [17]. In this study, we employ an in vitro model using 3D4/21 porcine alveolar macrophages exposed to DON in combination with LC-MS/MS-based non-targeted metabolomics. Our work expands our understanding of DON’s immunometabolism toxicity and offers potential therapeutic targets for mitigating mycotoxin-related risks in swine.

## 2. Materials and Methods

### 2.1. Cell Culture

The 3D4/21 cells were obtained from the American Type Culture Collection and grown in RPMI 1640 medium (HyClone, Marlborough, MA, USA) supplemented with 10% fetal bovine serum (FBS) and 1% penicillin–streptomycin solution (Gibco, New York, NY, USA). Cells were maintained at 37 °C with 5% CO_2_.

### 2.2. Cell Viability Assay

The 3D4/21 cells were seeded into 96-well plates at a density of 8 × 10^3^ cells/mL and treated with 0, 1, or 2 μM DON (Sigma-Aldrich, St. Louis, MO, USA) for 12, 24, or 48 h. Cell viability was assessed using the MTT assay (3-(4,5-dimethylthiazol-2-yl)-2,5-diphenyltetrazolium bromide; Beyotime, Shanghai, China) as previously described [17]. Briefly, 100 μL of fresh medium and 10 μL of freshly prepared MTT solution (5 mg/mL) were added to each well. After incubation, 100 μL of formazan solubilization solution was added to dissolve the crystals. The absorbance was measured at 570 nm using a microplate reader, and cell viability was calculated accordingly.

### 2.3. Metabolite Extraction

After treating 3D4/21 cells with or without DON (2 μM) for 24 h (six biological replicates per group), the cells were collected by centrifugation and stored in liquid nitrogen until further processing. The samples were first removed from liquid nitrogen, and 1 mL of −80 °C pre-cooled methanol was added to the sample containing 1 × 10^7^ cells. The mixture was vortexed for 30 s, then centrifuged at 4 °C and 13,000 rpm for 20 min. 1 mL of −80 °C pre-cooled HPLC-grade water was added to the remaining pellet, followed by one freeze–thaw cycle in liquid nitrogen, vortexed for 30 s, and centrifuged again (4 °C, 13,000 rpm, 20 min). Supernatants were pooled and centrifuged at 4 °C and 13,000 rpm for 20 min to ensure clarity. The clear supernatant was transferred to a fume hood and evaporated to dryness. The dried residue was reconstituted in 100 μL of initial mobile phase, sonicated for 5 min to ensure complete dissolution, and centrifuged at 4 °C and 13,000 rpm for 20 min prior to subsequent analysis.

### 2.4. Metabolite Identification and Data Analysis

Metabolite identification was performed using the LC-MS/MS by BIOMS Biotechnology (Beijing, China). Chromatographic separation was conducted on an ExionLC system (AB Sciex, Framingham, MA, USA) equipped with a Waters HSS T3 column (100 × 2.1 mm, 1.8 μM, Waters, Milford, MA, USA). Mass spectrometric analysis was carried out using a TripleTOF 5600 + system (AB Sciex) under both positive and negative ion modes with Information Dependent Acquisition (IDA) in high-sensitivity mode and dynamic background subtraction. In negative ion mode, mass spectrometry was performed with the following ion source parameters: sheath gas flow rate of 30 psi, Gas1 and Gas2 flow rates of 55 psi each, ion source temperature of 550 °C, and ion spray voltage of −4500 V. The data acquisition time was 14 min. The TOF MS scan range was set from *m*/*z* 100 to 1200, and each MS1 scan was followed by 12 product ion (MS/MS) scans. The MS/MS scan range was *m*/*z* 50~1200, with an accumulation time of 0.05 s for each MS/MS scan. The collision energy was set at −40 eV (spread ±20 eV). In positive ion mode, the same parameters were used except that the ion spray voltage was set at +5500 V and the collision energy was adjusted to +40 eV.

The raw data obtained from mass spectrometry detection were imported into Progenesis QI (v3.0) software for data preprocessing and metabolite identification. Metabolites were identified by referencing the Kyoto Encyclopedia of Genes and Genomes (KEGG, http://www.genome.jp/kegg/, accessed on 21 May 2024) and the Human Metabolome Database (HMDB, http://www.hmdb.ca, accessed on 21 May 2024). Quality control (QC) analysis was carried out to ensure the accuracy and reliability of the data. Metabolites with a coefficient of variation (CV) less than 30% in QC samples, a variable importance in the projection (VIP) value greater than 1 and a *p*-value less than 0.05 were selected as differential metabolites (DMs). To reveal the differences in metabolites among different components, principal component analysis (PCA) was carried out. To remove noise and identify metabolites that contribute most to group separation, orthogonal partial least squares discriminant analysis (OPLS-DA) was performed. The quality of the model was assessed by cross-validation, with R^2^Y and Q^2^ representing the explained variables and predictability of the model, respectively. Metabolic pathway enrichment was conducted using MetaboAnalyst (v5.0) (https://www.metaboanalyst.ca/, accessed on 27 May 2024) and KEGG (https://www.genome.jp/kegg/, accessed on 27 May 2024) to identify potentially dysregulated metabolic pathways.

### 2.5. Integrative Analysis of Metabolomics and Transcriptomics

Transcriptomic data were obtained from prior analysis of 3D4/21 cells treated with 2 μM DON for 24 h [17]. Differentially expressed genes (DEGs) filtered by log_2_ (fold change) ≥ 1 and FDR < 0.05. For metabolomics, 50 DMs with top VIP scores (VIP > 1) were selected, while 50 DEGs with the lowest FDR values were chosen for integration. Data were standardized (Z-score), and Pearson’s correlation coefficients (PCCs) between each DM-DEG pair were calculated in R (v4.2.1). Pairs with |PCC| ≥ 0.5 and *p* < 0.05 (adjusted by Benjamini–Hochberg) were considered significant. Pathway enrichment of significant pairs was performed via MetaboAnalyst. Correlation networks were visualized using Cytoscape (v3.9.1), with node sizes reflecting pathway enrichment and edge colors indicating positive/negative correlations.

### 2.6. Statistical Analysis

All experimental data are expressed as mean ± standard deviation (SD). Statistical significance was determined using one-way ANOVA followed by Tukey’s multiple comparisons test, with *p* < 0.05 considered significant. Different letters (e.g., a, b, and c) indicate statistically significant differences between groups.

## 3. Results

### 3.1. Viability of 3D4/21 Cells Following DON Exposure

**Figure 1:**
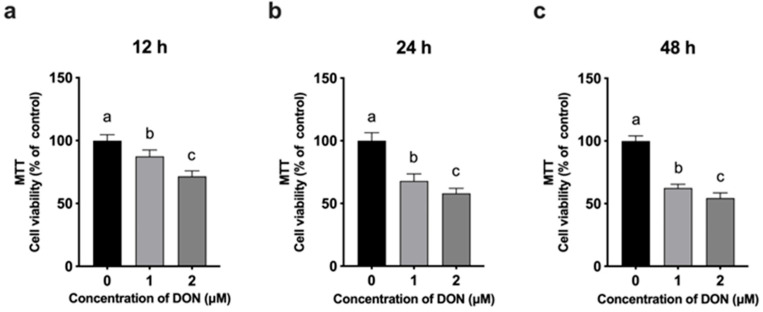
Effects of different concentrations of DON (0, 1, and 2 μM) on the viability of 3D4/21 cells assessed by MTT assay at 12 h (**a**), 24 h (**b**), and 48 h (**c**) after treatment. Data are presented as mean ± SD (*n* = 8). Different letters indicate significant differences among groups (*p* < 0.05, one-way ANOVA followed by Tukey’s multiple comparisons).

The cytotoxic effects of DON on 3D4/21 cells were evaluated using an MTT assay after treatment with 0, 1, and 2 μM DON for 12, 24, and 48 h. As shown in Figure 1a, DON exposure for 12 h led to a significant, dose-dependent decrease in cell viability. A similar trend was observed at 24 and 48 h (Figure 1b,c), with higher DON concentrations causing progressively reduced viability compared to the untreated control.

A concentration of 2 μM DON for 24 h was chosen for metabolomic analysis, as it caused significant cytotoxicity without excessive cell death, enabling the detection of biologically relevant metabolic changes. Furthermore, this concentration and time point were consistent with those used in our previous studies [17], ensuring comparability across experiments.

### 3.2. PCA Principal Component Analysis

**Figure 2:**
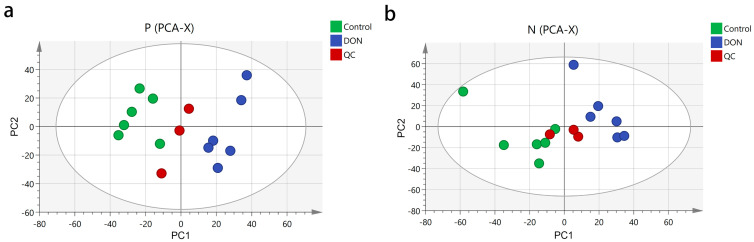
Principal component analysis score plot of the control and DON groups. Cells were treated with 2 μM DON for 24 h. (**a**) PCA score plot for the two groups analyzed in the positive ion mode. (**b**) PCA score pt for the two groups analyzed in the negative ion mode. PC1 was the first principal component; PC2 was the second principal component.

To investigate the metabolic characteristics of 3D4/21 cells after DON treatment, we conducted metabolomic profiling. Principal component analysis (PCA) showed distinct clustering patterns between the control and DON-treated groups, indicating significant metabolic perturbations induced by DON exposure. The peak areas of each metabolite in positive and negative ion modes were compared separately using SIMCA-P 14.1 software and subjected to principal component analysis. The three quality control (QC) samples were closely clustered near the center point in both the positive ion mode (Figure 2a) and the negative ion mode (Figure 2b). The samples of the same group were gathered in a relatively concentrated area and could be well distinguished from the other groups, confirming the reliability of the analytical method.

### 3.3. OPLS-DA Analysis and Iterative Validation

**Figure 3:**
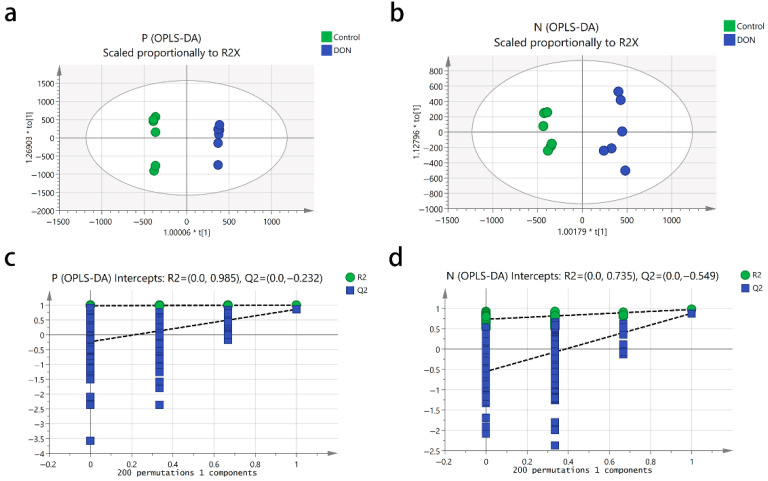
Orthogonal partial least squares discriminant analysis (OPLS-DA) score and permutation test plot in the positive ion mode (**a**,**c**) and the negative ion mode (**b**,**d**) for the control and DON groups. Cells were treated with 2 μM DON for 24 h. The intercept limit of Q^2^ was calculated by the regression line, which was the plot of Q2 from the permutation test in the OPLS-DA model.

To filter out orthogonal signals and establish the OPLS-DA model, we analyzed the metabolic differences between the control and DON-treated groups. The OPLS-DA score plots demonstrated clear separation between the two groups and tight clustering within each group (Figure 3a,b). In positive ion mode, the model achieved an R^2^Y of 0.99 and a Q^2^ of 0.86 (Figure 3a), while in negative ion mode, the R^2^Y and Q^2^ values were 0.97 and 0.88, respectively (Figure 3b). The OPLS-DA model quality was then validated through 200 iterations of cross-validation (Figure 3c,d). Permutation test results showed regression line intercepts for predictive ability at −0.232 (positive ion mode) and −0.549 (negative ion mode), confirming a well-fitting model without overfitting.

### 3.4. Metabolic Pathway Analysis

We identified 127 DMs between the control and DON treatment groups, using thresholds of CV < 30%, VIP > 1, and *p* < 0.05 (Table 1). Among these, 36 metabolites were upregulated and 41 metabolites were downregulated in the positive ion mode (Table S1), while 19 metabolites were upregulated and 26 metabolites were downregulated in the negative ion mode (Table S2).

The heatmap of differential metabolites revealed clear clustering between control and DON-treated groups in both positive (Figure S1) and negative ion modes (Figure S2), indicating that DON treatment significantly altered the metabolic profile of 3D4/21 cells. Furthermore, we ranked 127 differential metabolites by log_2_FC. In positive ion mode, ophthalmic acid, lysyl-Hydroxyproline and γ-Glutamylcysteinylserine were upregulated, while (±)-2-Methylthiazolidine, tyrosyl-Asparagine, valyl-Threonine, L-proline, and racemethionine were downregulated (Table 2). In negative ion mode, nitazoxanide, 5-Hexenyl glucosinolate, 2-Deoxy-6-O-sulfo-2-(sulfoamino)-D-glucopyranose, hypotaurocyamine and nicotinate D-ribonucleoside were upregulated, whereas nicotinic acid mononucleotide, asparagusic acid syn-S-oxide and gemcitabine were downregulated (Table 3).

**Figure 4:**
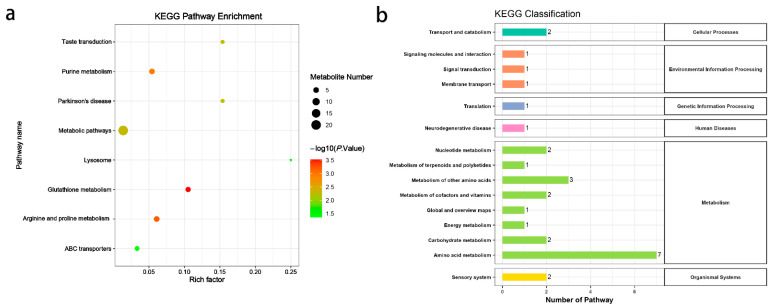
KEGG pathway enrichment analysis (**a**) and KEGG classification (**b**) of differential metabolites in 3D4/21 cells exposed to 2 µM DON for 24 h.

Enrichment analysis revealed that the differential metabolites were involved in eight metabolic pathways, including lysosomes, parkinsonism, taste transduction, purine metabolism, glutathione metabolism, arginine and proline metabolism, and ABC transporter proteins (Figure 4a). These pathways were classified under KEGG categories as follows: cellular processes (transport and catabolism); environmental information processing (signaling molecules and interaction; signal transduction; membrane transport); genetic information processing (translation); human diseases (neurodegenerative disease); metabolism (nucleotide metabolism; metabolism of terpenoids and polyketides; metabolism of other amino acids; metabolism of cofactors and vitamins; global and overview maps; energy metabolism; carbohydrate metabolism; amino acid metabolism); and organismal systems (sensory system) (Figure 4b).

### 3.5. Integration of Metabolomics Results with Transcriptomics

**Figure 5:**
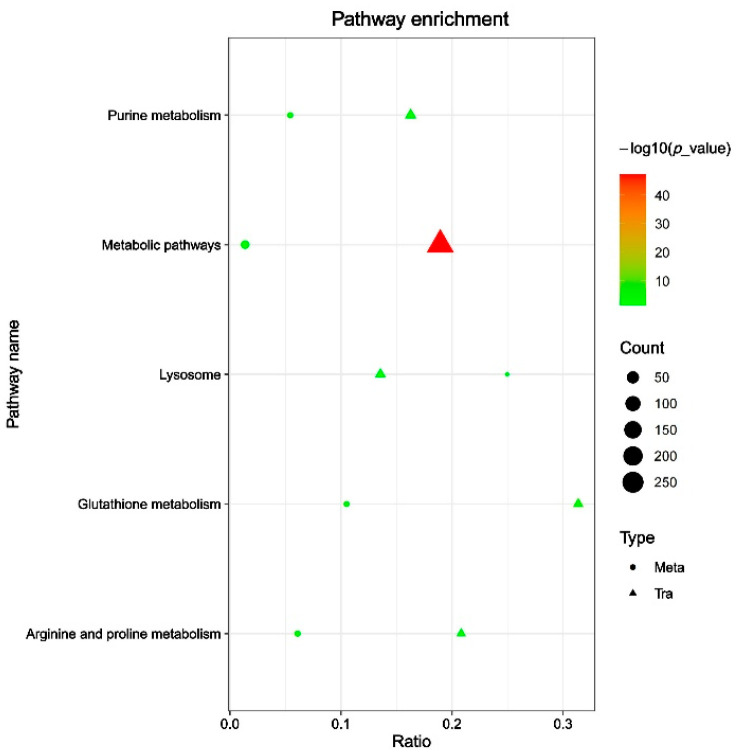
Integrated KEGG pathway enrichment analysis of metabolomic and transcriptomic data in 3D4/21 cells exposed to 2 µM DON for 24 h. Meta represents metabolomic data, and Tra represents transcriptomic data.

To provide mechanistic insight into links between gene expression and functional metabolic outcomes, we integrated our previous transcriptomic analysis of DON-induced 3D4/21 cells with current metabolomics data [17]. In the positive ion mode, specific correlation analyses (Figure S3) showed that the metabolite HMDB0039733 exhibited the strongest positive correlation with the gene *PDIA4*, with a correlation coefficient of 0.9994, while HMDB0039733 displayed the strongest negative correlation with the gene *TXNIP*, with a coefficient of −0.9989. Additionally, in the positive ion mode (Figure S4), the metabolite HMDB0125517 showed the strongest positive correlation with the gene *OTUD1* (0.9977), and the metabolite CSID54753 had the strongest negative correlation with the gene *BRCA1* (−0.9986). In addition, pathway enrichment analysis indicated that both differential genes and metabolites co-regulated purine metabolism, metabolic pathways, lysosome, glutathione metabolism, and arginine–proline metabolism (Figure 5).

## 4. Discussion

Deoxynivalenol (DON) is a well-known threat to porcine health and swine industry productivity because it damages macrophages. However, the global metabolomic perturbations underlying DON-induced immunotoxicity remain poorly understood. In this study, we employ non-targeted metabolomics, cell viability assays, and multi-omics integration to characterize the metabolic disturbances caused by DON in 3D4/21 cells. Our findings extend current knowledge of DON as a potent immunosuppressive mycotoxin in pigs.

The concentration- and time-dependent reduction in 3D4/21 cell viability corroborates our previous evidence of DON’s direct cytotoxic effects in 3D4/21 cells [17]. Consistent with our previous results, DON treatment led to a decrease in cell viability at 2 μM after 24 h exposure. Unexpectedly, a significant decrease also occurred at 1 µM after only 12 h, showing that DON exerts harmful effects even at low concentrations and that 3D4/21 cells are highly sensitive to it. In porcine alveolar macrophages (PAMs), 1 µM DON did not affect viability but did alter immune signaling [18]. Previous studies have shown that DON exerts variable effects depending on both the cell type and concentration. For example, in granulosa cells, low concentrations of DON have been reported to promote cell proliferation, potentially through enhanced IGF-I signaling, while higher concentrations inhibit proliferation, likely due to cytotoxic effects [19,20,21]. These findings underscore that the biological impact of DON is not only dose-dependent but also highly influenced by the cellular context, indicating that different cell types may respond to DON exposure via distinct mechanisms.

In our study, all samples are processed using a standardized methanol–water extraction protocol alongside QC samples. These QC samples clustered tightly in PCA plots, and all metabolites showed a CV below 30%, minimizing the likelihood of systematic errors such as ion suppression. Moreover, PCA and OPLS-DA analyses showed clear separation between control and DON-treated groups, indicating profound metabolic dysregulation [22]. The robust model fit (Q^2^ > 0.85) and absence of overfitting (permutation test results) validate the reliability of our metabolomic data, supporting the biological relevance of identified DMs.

DON drives oxidative stress management and rewires sulfur amino acid and NAD^+^ metabolism in porcine macrophages, metabolic axes closely tied to their inflammatory phenotype [23,24]. In positive ion mode, we observed elevated levels of ophthalmic acid, a sensitive proxy for glutathione depletion and oxidative stress [25], together with γ-glutamylcysteinylserine, a dipeptide that reinforces glutathione biosynthesis under inflammatory challenge [26]. Concomitant declines in racemethionine [27], L-Proline [28], and related dipeptides imply a constrained sulfur and aminoacid supply that supports macrophage methylation and stress-responsive metabolism. In negative ion mode, accumulation of nitazoxanide [29] points to an immunomodulatory signal, whereas the opposite shifts in nicotinate D-ribonucleoside (up) and nicotinic acid mononucleotide (down) reflect remodeling of the NAD salvage network that shapes macrophage effector programs [23]. Increased hypotaurocyamine [30] is consistent with taurine/hypotaurine antioxidative pathways, and the reduction in gemcitabine [31] aligns with lowered exposure to a nucleoside analog known for myeloid immunosuppression. Two high-abundance features were annotated as harzianopyridone, a pyridone alkaloid produced [32], and oxybutynin chloride, a compound with antimuscarinic activity [33]. As there is no evidence of endogenous synthesis of these compounds in macrophages or any mechanistic association with DON or PAMs, their observed increase is likely attributable to background contamination. This finding does not impact our conclusions regarding DON-induced immunotoxicity. These results emphasize the need for further validation to confirm compound identities and determine any residual biological relevance.

Through metabolic pathway analysis, we identified metabolic pathways associated with immune regulation. Glutathione (GSH) metabolism emerged as a critical pathway, with reduced GSH levels potentially compromising antioxidant defenses and exacerbating oxidative stress [34,35]. This aligns with DON’s known ability to induce reactive oxygen species (ROS) production and mitochondrial damage in porcine lymphocytes [10]. Depletion of GSH might disrupt redox balance, amplifying cellular injury and immune dysfunction [36]. Notably, our study reveals that disturbances in arginine and proline metabolism constitute another key pathway affected by DON exposure. Arginine is a critical substrate for both nitric oxide synthase (NOS) and polyamine synthesis—processes essential for macrophage effector functions [37]. Disruption of arginine metabolism by DON may therefore impair the production of immune-related proteins in 3D4/21 cells, potentially reducing their functional capacity. As alveolar macrophages, 3D4/21 cells serve as a primary defense against pulmonary pathogens [38,39]. Consequently, dysfunction in these cells due to altered arginine metabolism could weaken this critical immune barrier in pigs. In addition, perturbations in purine metabolism suggest impaired nucleotide synthesis and energy homeostasis, which may contribute to reduced cell viability and dysfunctional immune responses [40]. Elevated purine metabolites could reflect increased nucleic acid turnover due to cellular stress or apoptosis, which is consistent with extensive apoptosis observed in lymphocytes after DON exposure in pigs [41,42].

Integrated transcriptomic–metabolomic analysis highlighted joint enrichment of purine, glutathione, and arginine/proline pathways, implying that their interaction drives DON immunotoxicity. In piglet models, DON exposure causes thymic atrophy and lymphocyte apoptosis [43], which may be related to energy metabolism disorders (purine metabolism) and oxidative stress (glutathione metabolism) identified in this study. For example, GSH depletion can overproduce ROS and damage mitochondrial function [44], while purine metabolism disorders reduce ATP supply, leading to energy crisis in immune cells and accelerating apoptosis [45,46]. The metabolite HMDB0039733 (γ-glutamylcysteinylserine) correlated strongly with *PDIA4* and inversely with *TXNIP*, supported by stringent criteria (|PCC| ≥ 0.5, adjusted *p* < 0.05) and consistent patterns across replicates. PDIA4, a protein disulfide isomerase involved in endoplasmic reticulum (ER) stress responses [47], and TXNIP, a thioredoxin-interacting protein that modulates redox balance [48]. Their strong correlations indicate that *PDIA4* upregulation may counteract ER stress induced by DON [17], while *TXNIP* downregulation could attempt to relieve inhibition of thioredoxin, a key antioxidant enzyme, to compensate for glutathione depletion. *OTUD1* and *BRCA1* showed strong correlations with specific metabolites under stringent statistical thresholds. Functionally, *OTUD1* is involved in deubiquitination [49], while BRCA1 plays a key role in DNA repair [50], supporting their potential involvement in DON-induced toxicity. However, the specific roles of these genes in response to DON require further validation.

## 5. Conclusions

DON exposure significantly impairs 3D4/21 cell line viability and induces metabolic reprogramming. Key affected pathways include purine metabolism, glutathione metabolism, and arginine–proline metabolism. These disruptions suggest oxidative stress, energy imbalance, and immune dysfunction. Integrated metabolomic and transcriptomic analyses confirm their central role in DON-induced immunotoxicity. The study offers novel insights into DON’s effects on immune cells and identifies potential metabolic targets for mitigation.

## Figures and Tables

**Table 1:** Statistical results of differential metabolites.

Detection Mode	Differential Metabolomics		
	All	Up	Down
DON vs. Control (Negative mode)	45	19	26
DON vs. Control (Positive mode)	77	36	41

**Table 2:** Top 10 upregulated and top 10 downregulated metabolites after DON treatment in positive ion modes.

Compound Name	Formula	log_2_(FC)	Regulation
Harzianopyridone	C_14_H_19_NO_5_	6.89	Up
Dribendazole	C_15_H_19_N_3_O_2_S	5.07	Up
Ophthalmic acid	C_11_H_19_N_3_O_6_	4.7	Up
Lysyl-Hydroxyproline	C_11_H_21_N_3_O_4_	3.91	Up
γ-Glutamylcysteinylserine	C_11_H_19_N_3_O_7_S	2.65	Up
LENAMPICILLIN	C_21_H_23_N_3_O_7_S	2.55	Up
2-Acetylpyrrolidine	C_6_H_11_NO	2.4	Up
Gravacridonediol methyl ether	C_20_H_21_NO_5_	2.25	Up
3,11,12-Trihydroxy-1(10)-spirovetiven-2-one	C_15_H_24_O_4_	2.04	Up
Miserotoxin	C_9_H_17_NO_8_	2.00	Up
Oxybutynin Chloride	C_22_H_31_C_l_NO_3_	−4.83	Down
Coutaric acid	C_18_H_27_N_3_O_4_	−2.82	Down
(±)-2-Methylthiazolidine	C_4_H_9_NS	−2.24	Down
Cefminox	C_16_H_21_N_7_O_7_S_3_	−2.18	Down
THTC	C_5_H_8_O_2_S	−2.17	Down
Tyrosyl-Asparagine	C_13_H_17_N_3_O_5_	−2.15	Down
Valyl-Threonine	C_9_H_18_N_2_O_4_	−2.14	Down
L-Proline	C_5_H_9_NO_2_	−2.06	Down
Racemethionine	C_5_H_11_NO_2_S	−2.03	Down
2-O-alpha-D-Glucopyranosyl-D-glucopyranose	C_12_H_22_O_11_	−1.87	Down

**Table 3:** Top 10 upregulated and top 10 downregulated metabolites after DON treatment in negative ion modes.

Compound Name	Formula	log_2_(FC)	Regulation
Flurocitabine	C_9_H_10_FN_3_O_4_	4.76	Up
[(1-oxo-1H-isochromen-3-yl)methoxy]sulfonic acid	C_10_H_8_O_6_S	3.22	Up
Nitazoxanide	C_12_H_9_N_3_O_5_S	2.37	Up
5-Hexenyl glucosinolate	C_13_H_23_NO_9_S_2_	2.32	Up
Carbofenotion	C_11_H_16_ClO_2_PS_3_	2.23	Up
2-Deoxy-6-O-sulfo-2-(sulfoamino)-D-glucopyranose	C_6_H_13_NO_11_S_2_	2.05	Up
Hypotaurocyamine	C_3_H_9_N_3_O_2_S	1.95	Up
Nicotinate D-ribonucleoside	C_11_H_14_NO_6_+	1.87	Up
1-(6-Oxo-6H-benzo[c]chromen-3-yl)-1H-pyrrole-2,5-dione	C_17_H_9_NO_4_	1.73	Up
Methasulfocarb	C_9_H_11_NO_4_S_2_	1.66	Up
(2R,5Z)-4-Methyl-5-[2-(phosphonooxy)ethylidene]-2,5-dihydro-1,3-thiazole-2-carboxylic acid	C_7_H_10_NO_6_PS	−3.42	Down
2-(2,6-dihydroxy-3,4-dimethoxycyclohexylidene)acetonitrile	C_10_H_15_NO_4_	−2.79	Down
Nicotinic acid mononucleotide	C_11_H_15_NO_9_P	−2.78	Down
Flunidazole	C_11_H_10_FN_3_O_3_	−2.42	Down
Asparagusic acid syn-S-oxide	C_4_H_6_O_3_S_2_	−2.39	Down
Ethyl glucuronide	C_8_H_14_O_7_	−2.21	Down
MFCD00215956	C_20_H_11_NO_2_	−2.17	Down
DIAMIDAFOS	C_8_H_13_N_2_O_2_P	−2.06	Down
AY6315000	C_9_H_10_FNO_2_	−2.03	Down
Gemcitabine	C_9_H_11_F_2_N_3_O_4_	−1.96	Down

## Data Availability

The original contributions presented in this study are included in the article/Supplementary Materials. Further inquiries can be directed to the corresponding author(s).

## Supplementary Materials

The following supporting information can be downloaded at: https://www.mdpi.com/article/10.3390/ani15152324/s1. Table S1. The differential metabolites between the DON and control groups in the positive ion mode; Table S2. The differential metabolites between the DON and control groups in the negative ion mode; Figure S1. Heatmap of differential metabolites between the control and DON-treated groups in positive ion mode; Figure S2. Heatmap of differential metabolites between the control and DON-treated groups in negative ion mode; Figure S3. Correlation patterns between differentially expressed genes and differential metabolites (positive ion mode) in 3D4/21 cells exposed to 2 μM DON for 24 h; Figure S4. Correlation patterns between differentially expressed genes and differential metabolites (negative ion mode) in 3D4/21 cells exposed to 2 μM DON for 24 h.
