# Supplementary material for: Evaluation of Metabolic Characteristics Induced by Deoxynivalenol in 3D4/21 Cells
Source: Animals (Basel). 2025 Aug 7;15(15):2324. doi: 10.3390/ani15152324 (PMC12345648; doi:10.3390/ani15152324)
Supplement: Supplementary file 1 [file animals-15-02324-s001.zip › Supplementary Figures.pdf]

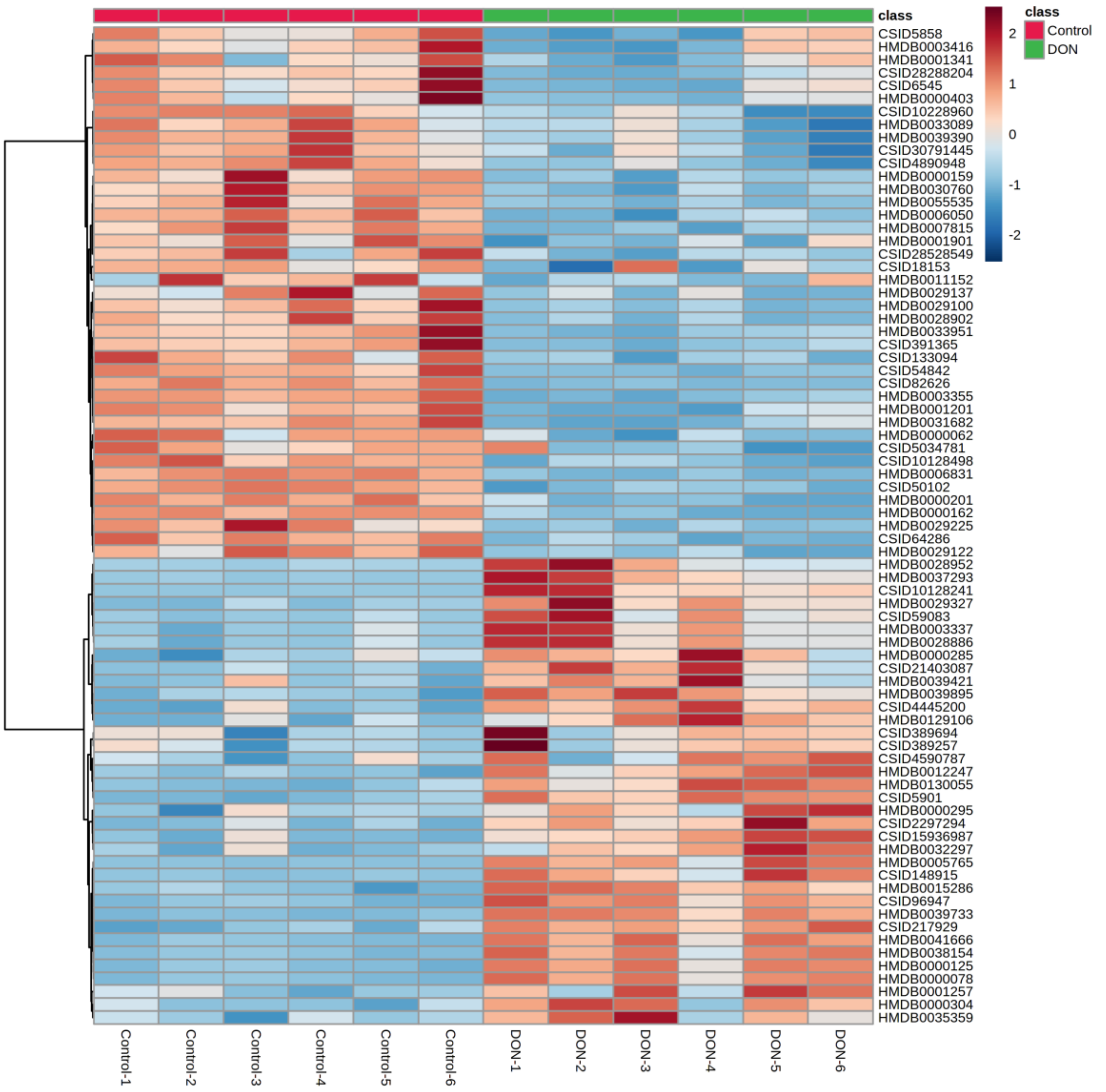

Figure S1. Heatmap of differential metabolites between the control and DON-treated groups in positive ion mode.

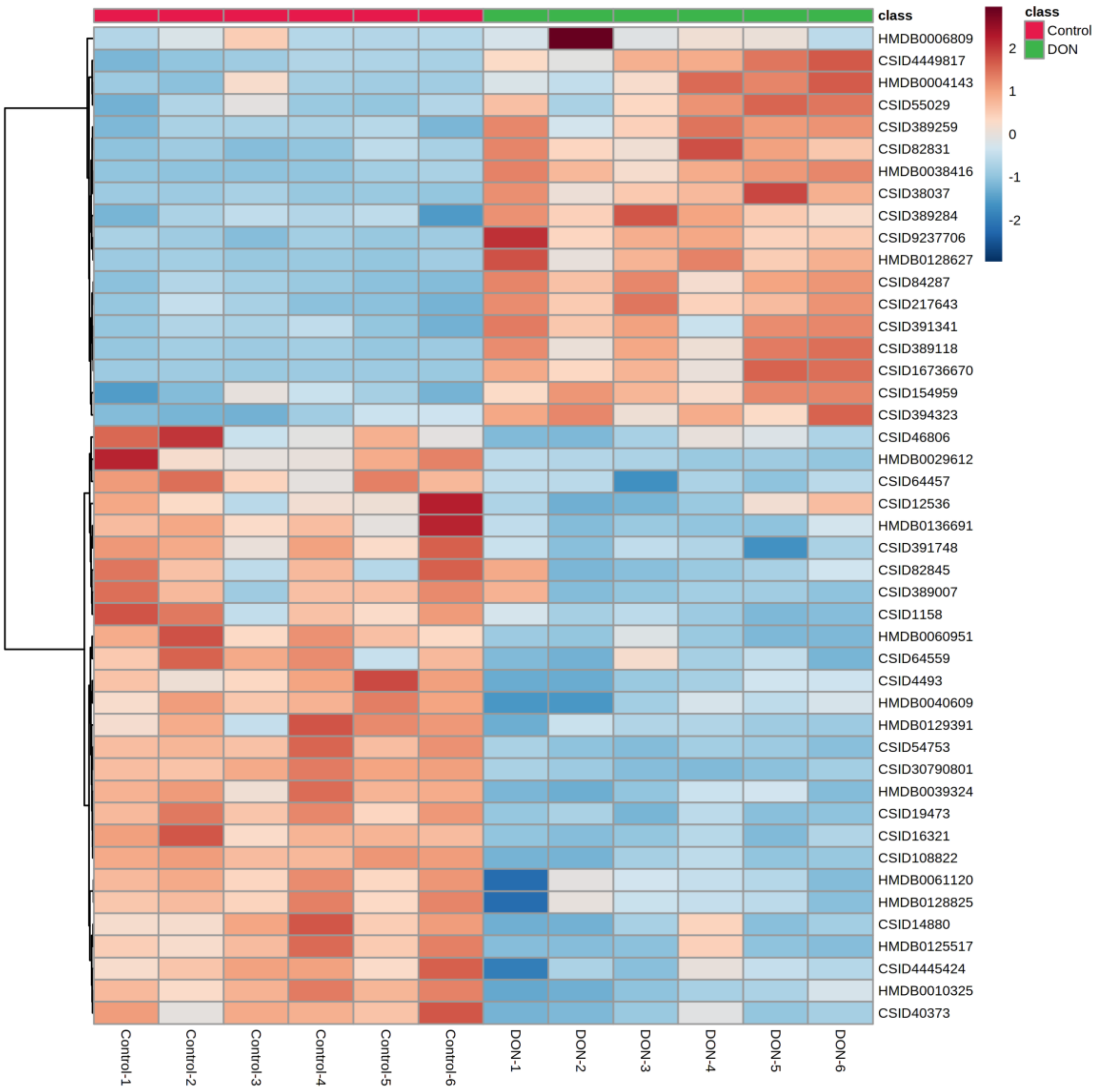

Figure S2. Heatmap of differential metabolites between the control and DON-treated groups in negative ion mode.

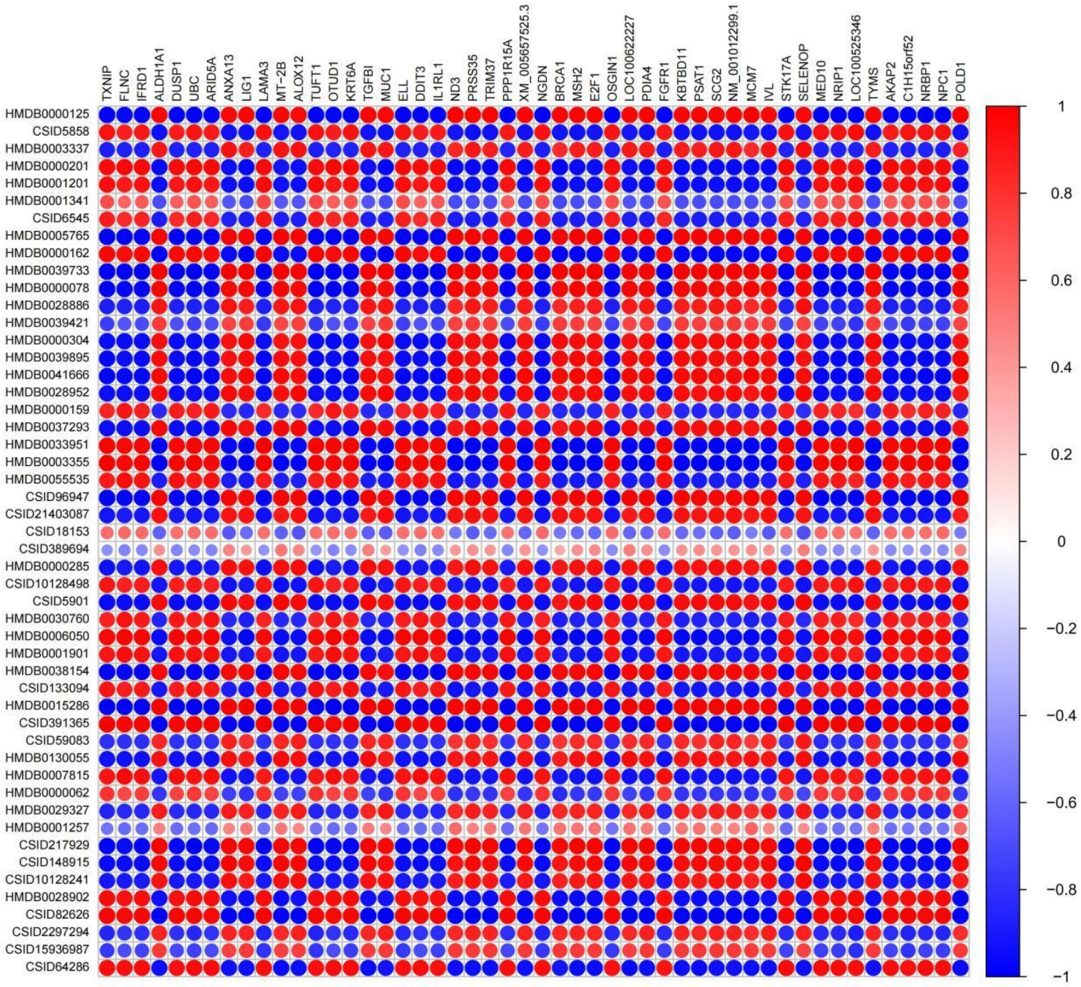

Figure S3. Correlation patterns between differentially expressed genes and differential metabolites (positive ion mode) in 3D4/21 cells exposed to 2 μM DON for 24 hours.

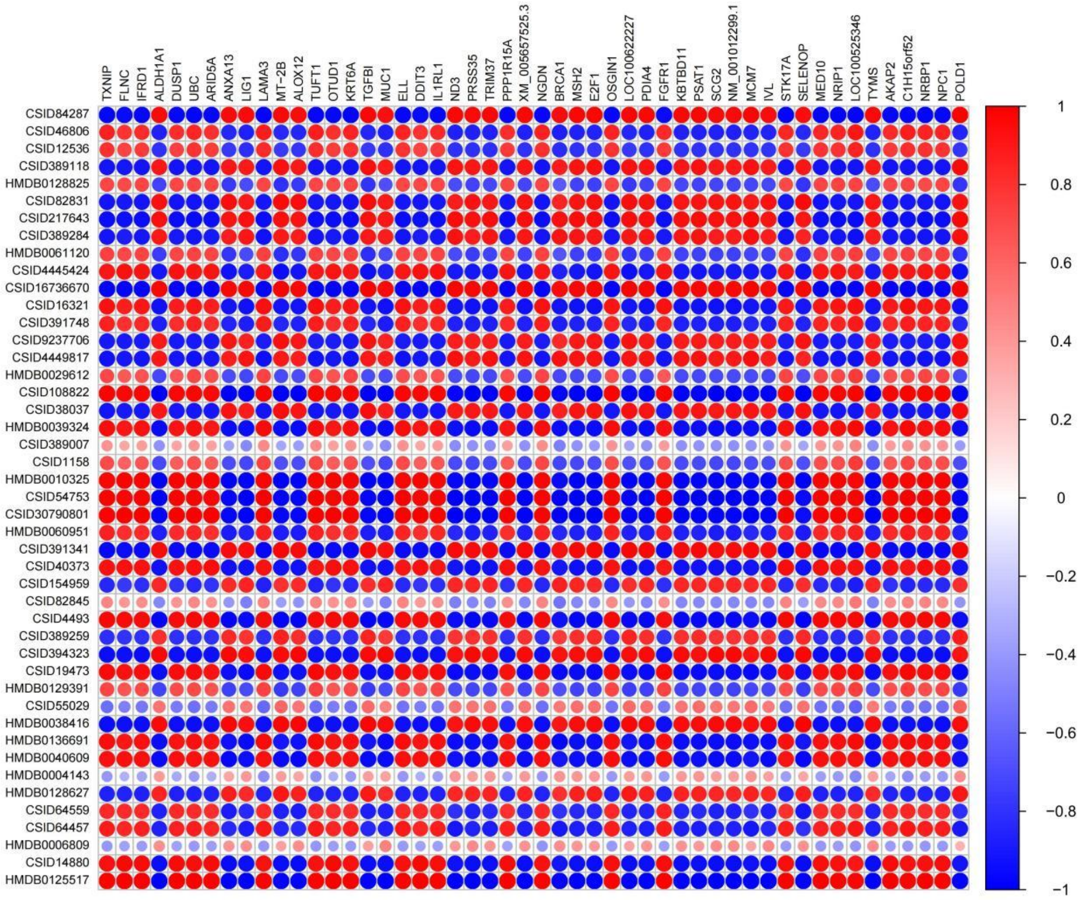

Figure S4. Correlation patterns between differentially expressed genes and differential metabolites (negative ion mode) in 3D4/21 cells exposed to 2 μM DON for 24 hours.
